# Ammonium Polyphosphate: Modification Strategies and Synergistic Flame-Retardant Applications

**DOI:** 10.3390/polym18141786

**Published:** 2026-07-21

**Authors:** Yina Liu, Rongjie Yang, Zhaolu Qin, Wenchao Zhang, Dinghua Li

**Affiliations:** National Engineering Research Center of Flame Retardant Materials, School of Materials Science and Engineering, Beijing Institute of Technology, Beijing 100081, China; 74201882025@bit.edu.cn (Y.L.); qinzhaolu@163.com (Z.Q.); zwc18@bit.edu.cn (W.Z.); dli@bit.edu.cn (D.L.)

**Keywords:** ammonium polyphosphate, modification, intumescent flame retardant, synergistic flame retardancy, flame-retardant mechanisms

## Abstract

Ammonium polyphosphate (APP) is widely used in intumescent flame-retardant (IFR) systems because of its environmental friendliness, low cost, and high flame-retardant efficiency. However, its practical applications are limited by high hygroscopicity, poor compatibility with organic substrates, and the high loading required in single-component systems. To address these limitations, extensive studies have been conducted on APP modification and synergistic flame-retardant systems. This review systematically summarizes the modification strategies and flame-retardant mechanisms of APP. The synergistic flame-retardant effects and mechanisms of APP combined with silicon-, boron-, and metal-containing compounds are also discussed. In addition, the effects on the flame-retardant performance of different structural characteristics, such as nanostructures, layered structures, and ring structures, are reviewed. Finally, the current challenges and future perspectives of APP-based flame-retardant systems are highlighted. This review provides useful guidance for the design, optimization, and practical application of advanced APP-based intumescent flame-retardant materials.

## 1. Introduction

Polymer materials are inherently flammable due to their chemical structure and composition, making them major contributors to ignition and fire propagation. With increasing demand for halogen-free and high-performance flame-retardant solutions, the limitations of traditional halogen-based, inorganic, and single-phosphorus flame-retardant systems have become increasingly evident. Consequently, intumescent flame-retardant (IFR) systems have emerged as a promising alternative. Halogen-free IFRs primarily rely on phosphorus and nitrogen additives. Their flame-retardant effects are caused by the synergistic interaction of three key components: an acid source, a carbon source, and a gas source. This mechanism allows IFRs to form a protective char layer that effectively insulates the underlying polymer, making them a critical strategy in the development of advanced flame-retardant polymer materials [1,2,3].

Of all the IFRs, ammonium polyphosphate (APP) has received considerable attention due to its environmentally friendly nature. APP generally refers to linear ammonium phosphates with a polymerization degree greater than 20, while ammonium phosphates with a polymerization degree between 2 and 20 are classified as oligophosphates. Early forms of APP by Schiff in 1857 through the reaction of phosphorus pentoxide with ammonia (NH_3_) [4] and synthesis methods became well-established during the 20th century. There are direct and indirect methods of APP synthesis; direct synthesis involves the high-temperature solid-state condensation polymerization reaction of phosphate salts (monoammonium phosphate [5,6,7,8,9,10], diammonium hydrogen phosphate [11,12,13,14], and triammonium phosphate [10]), P_2_O_5_, and urea at 200–350 °C. In addition, melamine can be used as an alternative nitrogen source [15]. In contrast, indirect synthesis occurs through phase transformation under controlled thermal conditions [16,17,18]. Currently, up to five or six crystal forms of APP have been reported, although the classification varies in the literature [19,20]. Types I, II, and V remain stable at room temperature, with V having the highest thermal stability and I the lowest [6,18,21,22]. Type I APP typically exhibits a polymerization degree below 100, with unit cell parameters of a = 14.50 Å, b = 24.59 Å, and c = 4.58 Å [18,23]. The unit cell parameters of Type II APP are a = 4.256 Å, b = 6.475 Å, and c = 12.04 Å [24]. Type II APP, with a high degree of polymerization (usually >1000), low water solubility (≤0.5 g/100 mL H_2_O), and excellent thermal stability, is the most widely used APP polymorph in flame-retardant applications. The differences in crystal structure and degree of polymerization among APP significantly influence their thermal stability, hydrolysis behavior, and compatibility with polymer matrices.

**Figure 1:**
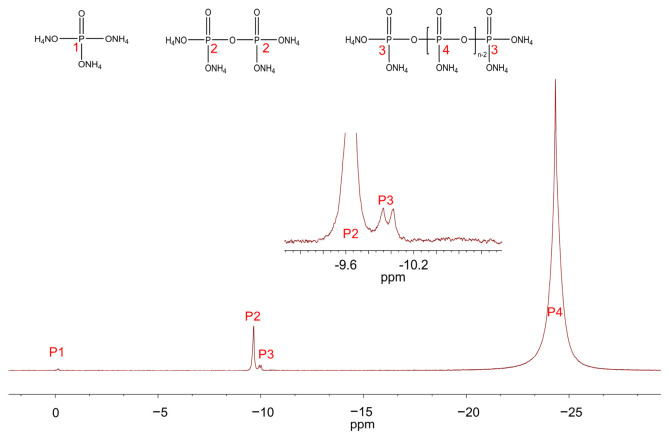
^31^P NMR spectrum of linear APP containing low-molecular-weight phosphate. The phosphorus sites labeled as 1, 2, 3, and 4 in the molecular structures correspond to the P1, P2, P3, and P4 signals in the ^31^P NMR spectrum, respectively. P1, P2, P3, and P4 are assigned to orthophosphate species, pyrophosphate species, terminal phosphate units, and internal phosphate units of linear APP, respectively.

In the infrared spectra of both Type I and II APP, the following absorption peaks arere present: the P=O bond vibration peak at 1250 cm^−1^, the P-O bond vibration peaks at 1070 cm^−1^ and 1010 cm^−1^, and the P-O-P bond vibration peak at 800 cm^−1^. Unlike Type II APP, Type I APP also exhibits characteristic absorption peaks at 760 cm^−1^, 680 cm^−1^, and 600 cm^−1^ [9,25]. In an aqueous linear APP solution at pH 6–7, liquid ^31^P NMR results (Figure 1) indicate that orthophosphate(P1) is calibrated at 0 ppm; the chemical shift of phosphorus bonded to one phosphorus(P2) lies between −8 ppm and −12 ppm, with coupled double peaks. In theory, the chemical shift of the phosphorus linked to two phosphates ranges (P4) should exhibit a triple peak. However, owing to the broad molecular weight distribution, a broad single peak was reported from −17 to −27 ppm in the ^31^P NMR spectrum [26,27,28]. On the basis of the above principles, ^31^P NMR analysis can be employed to characterize the polymerization degree of APP.

APP is widely used as a fertilizer, providing essential phosphorus and nitrogen to plants. It is harmless to humans, does not accumulate in food, and is degradable in both soil and sewage sludge [29]. Additionally, APP can form stable and soluble complexes with trace metal ions in soil through chelation, making it an effective fertilizer [30,31,32,33]. Since plant roots can only absorb water-soluble orthophosphate, phosphorus uptake depends on the kinetics of APP hydrolysis into orthophosphate and the extent of nutrient loss. Long-chain APP serves as a slow-release fertilizer agent [19,34]. Optimal fertilizer formulations can be achieved by balancing the rapid uptake of orthophosphate, fast hydrolysis of Type I APP, and slow hydrolysis of Type II APP, thus preventing nutrient loss and reducing the risk of eutrophication. In addition to its agricultural uses, APP has been applied as a fire-extinguishing agent. When dissolved in water and combined with salts, surfactants, wetting agents, stabilizers, thickeners, and foaming agents, APP functions as an aqueous fire suppressant suitable for various fire scenarios [35,36,37,38]. In its powder form, APP can be mixed with inorganic or organic compounds to extinguish oil and electrical fires [39,40,41,42]. Due to its fine powder particles, APP can also be combined with oxidizers, reducing agents, and binders to create both cold and hot aerosol extinguishing agents [43,44]. APP has also been used as a firebreak barrier in forests and as an explosion suppressant in coal dust explosions [45,46].

APP is most commonly used as a flame-retardant additive, with Type II APP experiencing relatively rapid development and application. Type II APP exhibits excellent thermal stability—with a decomposition temperature exceeding 270 °C—has low solubility, and produces no halogenated corrosive gas (e.g., HF, HCl, HBr) during combustion. It is widely used in green, environmentally friendly flame-retardant formulations and is extensively applied in polymers, coatings, wood, paper, and other materials [20]. As a key component of IFR, APP primarily serves as an acid and gas source. During the intumescent expansion process, the polymer melts when heated, and APP decomposes to produce NH_3_ and H_2_O, which may be partially trapped within the molten polymer. Concurrently, the phosphoric acid produced from APP decomposition reacts with hydroxyl or other synergistic groups to form unstable phosphate esters. These esters, upon dehydration, undergo carbonization, resulting in the formation of a foamy char layer [47]. Carbon sources can also be added to enhance the strength and quality of this char layer.

Although APP has been extensively investigated and widely utilized as a halogen-free flame retardant, challenges remain in achieving comprehensive structure–performance understanding and optimizing its flame-retardant efficiency. Previous studies have mainly focused on individual aspects of APP. For instance, Iben Hansen–Bruhn et al. [19] provided a detailed overview of the historical development, structure, synthesis methods, and applications of APP. In addition, several reviews have summarized the flame-retardant applications of APP in specific polymer matrices [47,48,49]. However, a systematic review of APP modification mechanisms and synergistic flame-retardant strategies involving various agents and structural architectures is still lacking.

Therefore, this review aims to systematically summarize APP modification strategies and high-performance APP-based flame-retardant systems, with emphasis on their synergistic flame-retardant mechanisms. First, APP modification strategies are classified according to their underlying mechanisms, and the resulting structural evolution is discussed. Subsequently, APP-based flame-retardant systems are summarized according to synergistic components and structural strategies. This review provides insights into the rational design of modified APP structures and the development of efficient, multifunctional, and sustainable APP-based flame-retardant materials.

## 2. Mechanism of Ammonium Polyphosphate Surface Modification

APP has attracted considerable attention as an efficient and environmentally friendly flame retardant due to its high phosphorus and nitrogen content. However, its practical application in polymer systems is still limited by several inherent drawbacks, including its partial water solubility, hygroscopicity, and poor interfacial compatibility with organic matrices. In addition, APP particles aggregate and migrate during processing, which further restricts their long-term stability and flame-retardant efficiency in polymer composites. Therefore, surface modification of APP has become an effective strategy for improving its interfacial compatibility and broadening its application range.

From a fundamental perspective, the surface modification of APP is governed by inter-facial interaction mechanisms between the phosphate species on the APP surface (including P=O, P-OH, and P-O^−^ groups) and functional groups of modifiers. These interactions can be generally categorized into three main types. The first is covalent bonding, where chemical reactions occur between reactive coupling agents and surface phosphate-related functionalities, leading to the formation of stable chemical bonds on the APP surface. The second is non-covalent molecular interactions, including electrostatic attraction and hydrogen bonding, which enable the adsorption and assembly of organic molecules or macromolecular species onto the APP surface without altering its intrinsic structure. The electrostatic interaction is primarily driven by the protonation of amino groups under neutral to acidic conditions and their subsequent coulombic attraction toward negatively charged phosphate species (P–O^−^), while hydrogen bonding further stabilizes the interfacial association. The third is coordination interaction, in which metal ions coordinate with oxygen-containing phosphate groups to form stable metal–phosphate complexes at the interface.

Modification methods can be categorized according to the interaction mechanism between the modifier and APP, i.e., covalent modification, non-covalent modification, and coordination modification (Table 1). These approaches differ significantly in their interfacial interaction mechanisms, modification efficiency, structural stability, and applicability. A clear understanding of these interaction mechanisms is essential for selecting suitable modification strategies and further optimizing the flame-retardant performance of APP. Therefore, the modification mechanisms and representative methods of each category are discussed in the following sections.

### 2.1. Covalent Coupling Modification

Silane coupling agents are widely employed as covalent surface modifiers for APP. In aqueous or alcohol–water media, organosilanes undergo hydrolysis to generate reactive silanol groups (Si–OH), which act as key intermediates governing subsequent interfacial reactions. These Si–OH groups can initially interact with phosphate groups and surface-oxygen-containing functionalities on APP through hydrogen bonding and electrostatic attraction, enabling their effective adsorption and homogeneous distribution on the particle surface.

**Figure 2:**
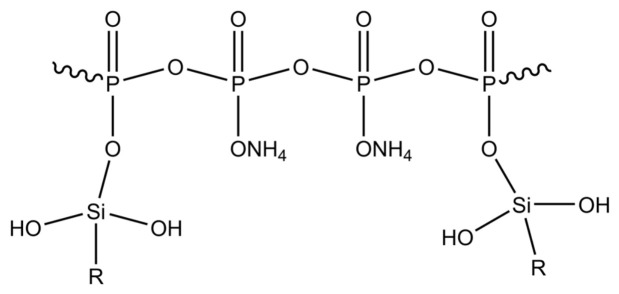
Structure of APP modified with ammonia-free silane coupling agents.

Upon further processing, condensation reactions occur between silanol groups and surface hydroxylated phosphate species, as well as between adjacent silanol groups. This leads to the formation of covalent Si–O–P linkages (Figure 2) and a crosslinked siloxane (Si–O–Si) network [50,51], which firmly anchors silane molecules onto the APP surface and establishes a stable interfacial layer at the molecular level. This modification process is fundamentally driven by hydrolysis–condensation chemistry, representing a typical covalent coupling mechanism at the solid–liquid interface.

**Figure 3:**
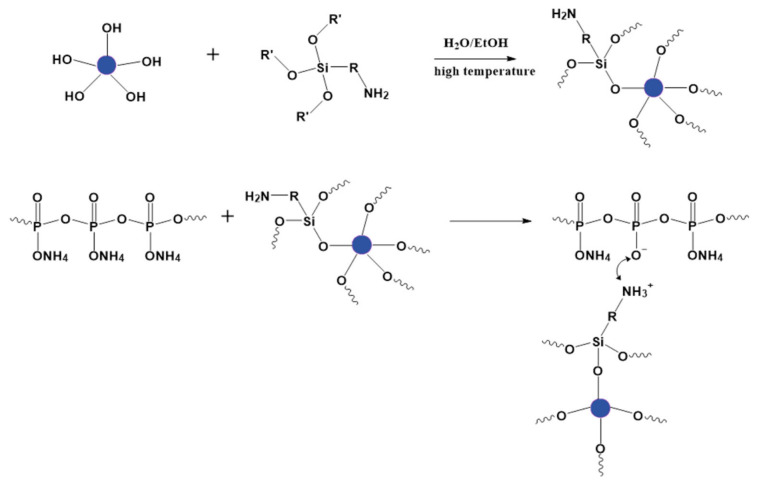
Schematic illustration of the silane coupling modification of inorganic nanoparticles (blue spheres) and their subsequent interaction with APP. The arrow indicates the proton transfer process between amino (–NH_2_) and hydroxyl (–OH) groups.

For amino-functional silane coupling agents, the presence of terminal –NH_2_ groups introduces an additional interfacial interaction pathway with APP. Under aqueous or slightly acidic conditions, these amino groups can be partially protonated to form –NH_3_^+^ species, which further enhances electrostatic attraction toward negatively charged phosphate groups on the APP surface, as illustrated in Figure 3. Representative studies have demonstrated the effectiveness of this strategy. For instance, Ou et al. [52] synthesized nano- silicon dioxide (SiO_2_) via a sol–gel method and subsequently functionalized its surface with γ-aminopropyltriethoxysilane (KH550), enabling its coupling with APP to form a micro/nano-structured P/N/Si hybrid flame retardant (APP@Si). Similarly, Feng et al. [53] grafted octa(propyl glycidyl ether)-functionalized polyhedral oligomeric silsesquioxane (POSS) onto the APP surface using KH550-mediated surface chemistry. In addition, KH550 treatment has been reported to significantly improve the particle size distribution and dispersibility of APP [51], indicating enhanced interfacial compatibility after surface modification.

The presence of hydroxyl and ammonium ions on the APP surface contributes to its strong hydrophilicity, resulting in a low contact angle of 19.9°. After modification with N-β-(aminoethyl)-γ-aminopropyltrimethoxysilane and carbon black (CB), the surface NH_4_^+^ species were partially replaced by CB, leading to the formation of carbon-black-modified APP (CBAPP), which exhibited a significantly increased contact angle of 77.9°, indicating markedly improved hydrophobicity [54].

Overall, silane coupling modification provides an effective covalent strategy to tailor the surface chemistry of APP and improve interfacial interactions with functional modifiers, thereby enabling the construction of well-designed hybrid flame-retardant systems.

### 2.2. Non-Covalent Interactions

#### 2.2.1. Amino-Functional and Electrostatic Interactions

**Figure 4:**
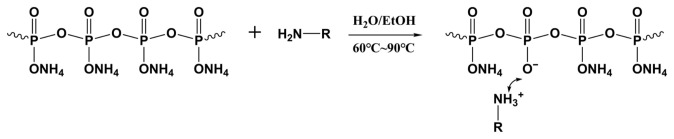
Modification of APP using amino-functional organic compounds. The arrow indicates the proton transfer process between amino (–NH_2_) and hydroxyl (–OH) groups.

Amino-functional modifiers interact with APP primarily through electrostatic attraction between protonated amino groups and phosphate species, accompanied by hydrogen-bonding interactions. Such interactions facilitate the immobilization of carbon-rich organic compounds on the APP surface without altering its intrinsic structure. As shown in Figure 4, amino-functional organic compounds have been employed to functionalize APP, enabling the direct introduction of carbon-rich moieties onto its surface. This strategy facilitates the development of highly efficient IFRs that integrate acid, carbon, and gas sources within a single architecture.

Studies have confirmed this interaction mechanism. Hai Thi Doan [55] synthesized polyethyleneimine (PEI)-modified APP (APP@PEI) via a solution treatment of APP with PEI in ethanol and deionized water at 90 °C. APP@PEI particles are effectively dispersed within the epoxy resin (EP). matrix. This is attributed to the compatibility of APP@PEI with EP Similarly, Zou et al. [56] and Shao et al. [57,58,59] modified APP using various amine compounds, including isopropylamine, ethanolamine, ethylenediamine, and diethylenetriamine. These treatments introduce carbon-containing amine compounds, leading to superior flame-retardant performance compared with the pristine APP. The water solubility of APP was reduced after being modified by ethylenediamine [58]. Nevertheless, such modifications tend to decrease the thermal decomposition temperature of APP [80], which may adversely affect its thermal stability during processing.

#### 2.2.2. Hydrogen-Bonding-Dominated Interactions

**Figure 5:**
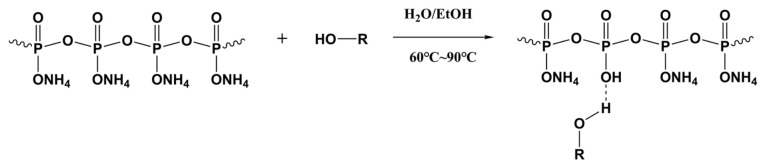
Modification of APP with hydroxyl-containing materials. The dashed lines indicate hydrogen bonding interactions.

Hydrogen-bonding-dominated modification is an important non-covalent strategy for the surface functionalization of APP. The abundant phosphate groups on the APP surface, including P=O and P–OH moieties, can act as effective hydrogen bond acceptors and donors, enabling strong intermolecular interactions with modifiers containing hydroxyl, amino, or carboxyl groups. Through these interactions, modifier molecules can be adsorbed and assembled onto the APP surface, forming a stable interfacial layer without altering the intrinsic chemical structure of APP. Moreover, this interaction mechanism provides an effective route for the utilization of naturally derived macromolecules rich in hydroxyl (R-OH) in APP surface modification (Figure 5).

Among these hydroxyl-containing modifiers, chitosan (CS), a naturally abundant polysaccharide rich in hydroxyl and amino functional groups, has been widely employed to construct core–shell-structured APP-based flame retardants. Typically, APP@CS is prepared via a solution treatment in ethanol–water systems at elevated temperatures, where interactions between the hydroxyl groups of CS and NH_4_^+^ ions of APP facilitate the formation of a bio-based coating layer [60,61]. It has been reported that the decomposition temperature (T_10_%) of APP@CS is slightly lower than that of pristine APP [61]. However, when incorporated at low loadings into polymer matrices, APP@CS can promote char formation during combustion, thereby enhancing the overall flame-retardant performance.

However, the strong hydrophilicity of CS limits performance at higher loadings by increasing the melt flow index of hygroscopic polymers, potentially causing dripping during combustion [63]. To address this, SiO_2_-modified APP@CS (APP@CS@SiO_2_) has been developed via self-assembly techniques, effectively reducing water absorption and improving overall stability [64,65]. Structural optimization has also been achieved by introducing additional gas-generating components. For example, a hierarchical microencapsulated flame retardant, (MF)CS-APP, uses APP@CS as the core and melamine–formaldehyde (MF) resin as the outer shell. Incorporation of (MF)CS-APP into PU matrices enhances flame retardancy while improving interfacial compatibility and mechanical properties, including tensile strength and elongation at break [64].

Bio-based lignin (LG) has attracted attention due to its UV absorption capability arising from phenolic hydroxyl groups. Sequential deposition of CS and LG onto APP constructs a hierarchical core–shell structure (APP@CS@LG), providing UV-shielding functionality. When incorporated into polylactic acid (PLA), this system blocks up to 99% of UV radiation, significantly improving polymer aging resistance [67]. LG has also been employed as a single modifier. Song et al. [68] developed LG@APP via hydrogen bonding between phenolic/aliphatic hydroxyl groups of LG and APP. At 6 wt% loading, the resulting EP composite achieved a UL-94 V-0 rating, with a 63.5% reduction in the peak heat release rate (pHRR) and a 51.3% decrease in the peak smoke production rate (pSPR). LG@APP promotes the formation of a compact char layer, enhancing fire protection, while the LG shell improves interfacial compatibility. Mechanical properties (including tensile strength, flexural strength, and impact strength) are improved to 50.8 MPa, 86.1 MPa, and 7.81 kJ/m^2^, respectively, surpassing the neat EP (48.3 MPa, 81.5 MPa, and 7.45 kJ/m^2^). Additionally, the phenolic and methoxy groups in LG impart antibacterial functionality, expanding application potential [69].

**Figure 6:**
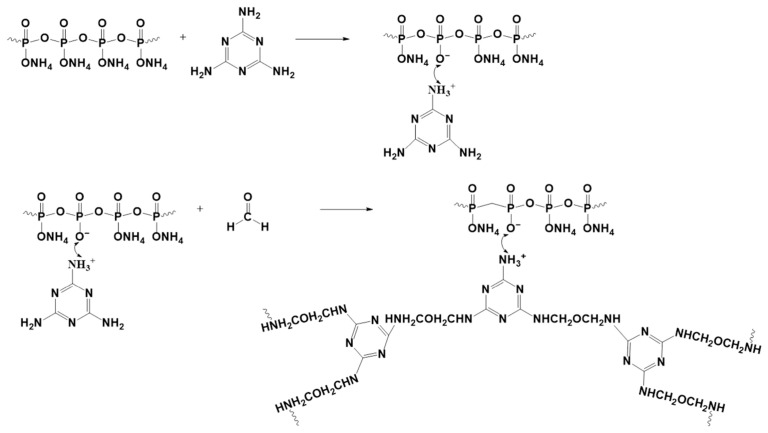
Modification process of MF resin-modified APP. The arrow indicates the proton transfer process between amino (–NH_2_) and hydroxyl (–OH) groups. The arrow indicates the proton transfer process between amino (–NH_2_) and hydroxyl (–OH) groups.

Non-covalent interactions can also induce the interfacial assembly of polymeric systems such as MF, urea–formaldehyde (UF), and polyurethane (PU) [81]. In these systems, hydrogen bonding and electrostatic interactions between APP surface phosphate groups and reactive functional groups (e.g., −NH_2_, −OH) initiate adsorption and subsequent in situ polymerization, leading to the formation of a continuous coating layer. MF resin is the most extensively employed encapsulation material due to its high nitrogen content and thermally stable triazine ring structure. The modification mechanism of MF-coated APP is illustrated in Figure 6. In this process, APP is introduced during the in situ polymerization of MF, serving as a reactive substrate. Interactions between hydroxyl groups on the APP surface and the -NH_2_ groups of melamine (MEL) facilitate the in situ formation of MF on the APP surface, resulting in MFAPP. MEL, as the key component of the IFR system, plays a dual role by acting as a gas source and reinforcing the structural integrity of the char layer. Since 2008, MEL-based resins and their derivatives have been widely utilized to modify APP, leveraging their intrinsic nitrogen-rich structure and thermal stability to enhance flame-retardant performance. MF-microencapsulated APP has been successfully applied in various polymer matrices, including polypropylene (PP) [82,83], ethylene-vinyl acetate (EVA) [84], ethylene propylene diene monomer [85], and PU [70]. Compared with the pristine APP, these systems exhibit reduced water absorption [47]. However, MF-coated APP inherently lacks a sufficient carbon source and therefore typically requires the incorporation of additional organic intumescent agents to achieve optimal performance [57]. However, the improvement in hydrophobicity of MF-modified APP is limited, and further surface treatment using silane coupling agents can be employed to enhance it [71,72,73]. Due to the inherent toxicity and environmental concerns associated with MEL, its application has been increasingly restricted by regulatory policies in many countries.

### 2.3. Coordination Interactions

The phosphate groups on the surface of APP, including P=O, P–OH, and P–O^−^ species, are rich in oxygen atoms that can act as effective coordination sites for metal ions. In a water and ethanol mixed-solvent system, multivalent metal ions (e.g., Fe^3+^, Zn^2+^, Co^2+^) can be enriched on the APP surface through electrostatic interactions and ion adsorption. Subsequently, introduced organic ligands containing multidentate nitrogen- or oxygen-donor groups can coordinate with the adsorbed metal ions, thereby inducing the directional assembly of metal centers and forming primary metal–ligand coordination units.

**Figure 7:**
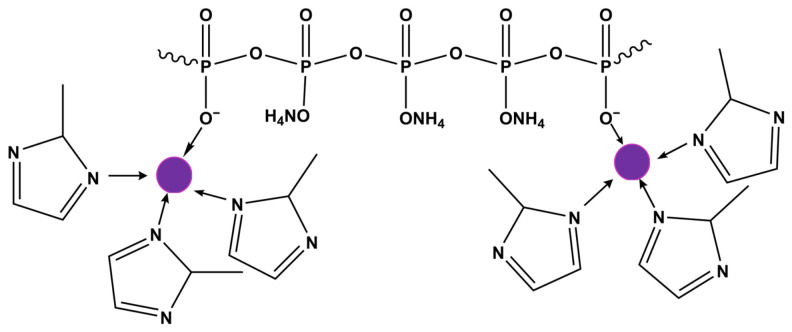
Schematic illustration of the coordination interaction between APP and ZIF-67, where the purple spheres represent Co^2+^ metal nodes of ZIF-67. The arrow indicates the metal ions coordinate with oxygen-containing phosphate groups.

With the progression of the coordination process, these primary units progressively aggregate and extend, leading to the formation of continuous metal–organic coordination networks (MOFs), such as ZIF-type MOFs, on the APP surface. This process is essentially a self-assembly behavior driven by three sequential stages, namely interfacial ion enrichment, coordination unit formation, and network growth. In this system, metal ions serve as coordination nodes, while organic ligands provide bridging and structural extension, enabling the transformation from molecular-scale coordination species to ordered two-dimensional or three-dimensional architectures. Based on this mechanism, APP surface modification can be achieved by anchoring different metal ions, such as Fe^3+^ [74], Zn^2+^ [75], Co^2+^ [76], and Cu^2+^ [77], followed by the introduction of various organic ligands to tailor the interfacial structure. The incorporation of organic components can further improve the interfacial compatibility between APP and organic matrices. As shown in Figure 7, APP modified with Co^2+^ and imidazole ligands is presented as a representative example. In addition, layered double metals can also be constructed on the APP surface via a two-step synthesis strategy [78]. Further examples of MOF-modified APP and their synergistic flame-retardant applications are discussed in Section 3.1.3.

## 3. APP Synergistic Flame Retardants in Polymer Materials

### 3.1. Elemental Synergistic Systems

#### 3.1.1. Silica-Containing Compounds

Silica-containing compounds have attracted considerable attention as synergistic additives for APP because of their high thermal stability, low cost, natural abundance, and ability to reinforce the condensed-phase protective layer during combustion [86,87]. Depending on their chemical composition and structure, silicon-containing synergists can be broadly classified into inorganic silicate fillers (e.g., kaolinite, wollastonite, vermiculite (VM), and SiO_2_) and organosilicon modifiers. Although both categories improve the flame retardancy of APP-based systems, their synergistic mechanisms differ substantially. Inorganic silicates mainly enhance the barrier effect of the char layer, whereas organosilicon modifiers additionally improve the interfacial compatibility between APP and polymer matrices.

The synergistic effect of kaolinite has also been demonstrated in other polymer matrices. As summarized in Table 2, the incorporation of kaolinite generally increased the limiting oxygen index (LOI) from 34.0% to 39.5% in PP, whereas reductions in total heat release (THR) were limited (3~4%). In contrast, smoke suppression was considerably more pronounced, with the total smoke production decreasing by up to 15%~45%. These results indicate that kaolinite primarily functions as a condensed-phase synergist by reinforcing the integrity of the intumescent char and suppressing volatile release rather than substantially reducing heat release. Nevertheless, its influence on mechanical properties remains strongly dependent on the polymer matrix, suggesting that further improvement in interfacial compatibility is still required.

This synergistic strategy has also been extended to other silicate minerals. For example, Quach et al. [93] incorporated wollastonite together with APP into polystyrene (PS). During combustion, APP promoted char formation, while phosphorus–silicate interactions facilitated the development of a compact and thermally stable protective layer composed of carbonaceous char, calcium-containing species, and phosphosilicate structures, thereby effectively suppressing the release of combustible volatiles.

Similarly, Barczewski et al. [94] reported that low-cost silicon-containing fillers, including expanded VM, copper slag, and basalt powder, could significantly reduce heat release by promoting the formation of ceramic-like protective char layers. However, the flame-retardant efficiency of these fillers was strongly dependent on loading level. In the PE/APP system, at low filler contents (5–10 wt%), the reductions in the heat release rate (HRR) and total heat release (THR) were small, and in some cases a slight increase in heat release was observed. When the filler content was increased to 20 wt%, a more pronounced reduction in HRR was achieved, indicating improved barrier effectiveness at higher loadings.

Nevertheless, these silicate fillers generally led to an increase in smoke production. Among the investigated systems, VM exhibited the most effective synergistic flame-retardant performance with APP at identical loadings, as evidenced by a significant reduction in the combustion rate and the formation of a more stable and continuous protective barrier. However, all three fillers adversely affected the mechanical properties of polyethylene (PE), primarily due to poor interfacial compatibility with the polymer matrix, resulting in weak interfacial adhesion and stress concentration effects.

Among the inorganic silicon-containing synergists, SiO_2_ has been extensively investigated owing to its excellent thermal stability, non-combustibility, and barrier effect at elevated temperatures. Zvonimir Katančić et al. [95] incorporated SiO_2_ and APP into PS by using melt blending. Although the addition of SiO_2_ did not significantly increase the LOI, it improved the thermal stability of PS and partially mitigated the deterioration in tensile strength induced by APP. Meng [96] further demonstrated that the flame-retardant performance of SiO_2_ is strongly dependent on its morphology. At a constant APP/pentaerythritol (PER) loading of 20 wt%, the incorporation of nano-, micro-, and hollow-structured SiO_2_ at 0.25 wt% resulted in LOI increases of 2.5%, 3.7%, and 7.0%, respectively, with the hollow SiO_2_ system achieving the highest LOI of 26.3%. This improvement was attributed to its higher specific surface area and hollow architecture, which enhance thermal insulation and mass transfer resistance during combustion. However, a further increase in SiO_2_ content led to a decrease in LOI, indicating a clear non-linear relationship between loading level and flame-retardant efficiency. Meanwhile, the reduction in heat release remained relatively limited across all formulations, suggesting that SiO_2_ primarily functions as a physical barrier and thermal insulator rather than an active flame-inhibiting agent. This highlights the inherent limitation of SiO_2_-based systems, where performance is highly morphology-dependent but difficult to further optimize through simple content adjustment.

A comparison of the reported inorganic silicon-containing synergists reveals distinct structure–property relationships. Layered silicates, such as kaolinite and VM, generally exhibit stronger synergistic effects than conventional SiO_2_ because their lamellar structures facilitate the formation of continuous ceramic-like barrier layers during combustion. In particular, VM exhibited the most pronounced improvement in flame retardancy among the reported mineral fillers, whereas kaolinite was more effective in suppressing smoke evolution. By contrast, SiO_2_ mainly contributed to thermal stabilization and only marginally reduced heat release, indicating that particle morphology is more important than simply increasing filler loading. Moreover, although inorganic silicate fillers effectively reinforce the condensed-phase barrier, their poor compatibility with nonpolar polymers frequently leads to deterioration of mechanical properties. Therefore, improving interfacial interactions while maintaining the barrier effect remains a major challenge for the development of inorganic silicon-containing APP synergists.

Therefore, recent research has increasingly focused on enhancing interfacial compatibility through chemical modification. Silicon, as an effective flame-retardant element, exhibits strong synergistic effects with APP, and surface modification using organosiloxane compounds—particularly silane coupling agents—has emerged as a promising strategy.

For example, Hao et al. [97] modified APP using amino-functional silane coupling agents with varying amino contents and used dipentaerythritol (DPER) as the carbon source. The modified APP showed a reduced initial decomposition temperature and an extended degradation range, which are favorable for intumescent systems. When incorporated into PP at 20 wt% (modified APP:DPER = 3:1), the composite exhibited a 6% increase in LOI, along with enhancements of 9% in tensile strength and 71% in elongation at break compared with unmodified APP/DPER, suggesting improved interfacial compatibility. Alternative siloxane-based strategies have also been explored. Phenyltrimethoxysilane (PTMS) was used as a precursor to encapsulate APP via a sol–gel process, yielding organosilane-coated APP (MAPP) [98]. Compared with pristine APP, MAPP exhibited significantly improved high-temperature thermal stability, with the initial decomposition temperature increasing from 221 °C to 338 °C. However, its flame-retardant performance showed no significant improvement, despite the enhanced thermal stability. Further advancements were reported by Ke [99], who prepared a self-crosslinkable vinyl polysiloxane coating on the APP surface. The resulting PP composites exhibited improved water resistance, flame retardancy, and mechanical performance. Notably, after the water resistance test, the deterioration in flame-retardant performance was significantly reduced, indicating enhanced environmental durability of the modified system. A reduction of 84.2% in the interfacial energy between APP@PD and PP was also observed, confirming a substantial improvement in interfacial compatibility. At elevated temperatures, the synergistic interaction between silicon and phosphorus promoted the formation of a dense and stable char layer containing Si–O–Si and Si–O–P structures, thereby enhancing thermal stability and improving the integrity of the protective barrier.

Table 3 shows a comparison of different silicon-containing synergists for APP-based systems. Overall, silicate minerals enhance condensed-phase barrier formation and smoke suppression but suffer from poor compatibility and mechanical drawbacks. SiO_2_ acts primarily as a thermal insulating filler with morphology-dependent and limited flame-retardant efficiency. In contrast, organosiloxane modifiers provide more balanced improvements in interfacial compatibility, char quality, and durability, although their application is restricted by synthesis complexity and scalability issues.

These differences indicate that no single silicon-based system can simultaneously optimize all performance aspects. Therefore, future studies should focus on integrating their complementary functions into hybrid systems for enhancing synergistic flame retardants.

#### 3.1.2. Boron-Containing Compounds

Boron-containing compounds exhibit characteristic thermal behavior, a softening at elevated temperatures to form boron-rich glassy protective layers that effectively suppress the release of flammable volatiles and inhibit heat and mass transfer. High-temperature conditions can cause polymer crosslinking, particularly in hydroxyl-containing systems, leading to the formation of thermally stable glassy ester structures. Various strategies have been developed to integrate boron species with APP, including (1) physical blending of boron compounds with APP [100,101,102,103]; (2) incorporation of boron into charring agents to enhance synergistic interactions with APP; (3) surface modification of APP using boron- and amino-functionalized compounds; and (4) synthesis of boron-containing APP derivatives.

Kumar et al. [104] incorporated nano-SiO_2_ and APP as flame-retardant additives together with boric acid/borax-modified bamboo fibers into PE. The combined system effectively promoted the formation of a compact char layer, which acted as a thermal barrier and improved resistance to heat transfer [105].

Zheng et al. [106] devised a boron–phosphorus synergistic system by functionalizing octadecyl alcohol with fatty alcohols and boric acid, followed by incorporation into expanded graphite and APP-based PE composites. During combustion, APP decomposes to release H_2_O and NH_3_, whereas boric acid undergoes dehydration to form B_2_O_3_, which further reacts with phosphoric intermediates generated from APP decomposition, leading to the formation of boron phosphate (BPO_4_). A dense crosslinked char structure is consequently generated, effectively suppressing smoke production. In addition, boron oxide was observed in the inner char region and was gradually converted into boron phosphate at the outer layer, resulting in a highly consolidated and partially graphitized protective residue. This phosphorus–boron synergy enhances char expansion and crosslinking density. Moreover, the release of water vapor from boric acid decomposition contributes to the dilution of combustible gases, while boron oxide forms a glassy layer that further blocks heat and oxygen diffusion above 450~500 °C. Compared with boric acid alone, the presence of APP significantly enhances the synergistic effect, leading to a 101% increase in char residue. The UL-94 rating is improved from no rating to V-2, and ignition delay is extended by 54 s, demonstrating substantially improved flame-retardant efficiency.

Chemical modification strategies further improve the performance of APP-based systems. Polyborosiloxane (BSi) has been used to microencapsulate APP, forming a P–B–Si hybrid shell structure (BSi-APP). This architecture is expected to suppress flame propagation and mass transfer while promoting char formation through enhanced decomposition of APP into phosphoric acid and subsequent crosslinking reactions [107]. In addition, the polysiloxane component improves interfacial compatibility with polymer matrices, thereby enhancing mechanical performance. However, experimental results indicate that the improvement in flammability and the LOI is not always significant at lower loading levels, suggesting that flame-retardant efficiency is strongly dependent on additive content. At 7 wt% loading, PLA containing BSi-APP (5 wt%) achieves a UL-94 V-0 rating, with the LOI increasing from 26.7% to 28.5%.

Wu et al. [108] synthesized a boron-containing APP (B-APP) via an ion-exchange-assisted process involving amine proton transfer between APP and 3-aminophenyl boronic acid. Compared with PLA/5% APP, the PLA/5% B-APP composite exhibited an increased LOI from 27.2% to 29.4% and an improved UL-94 rating from V-2 to V-0. Meanwhile, the THR decreased from 78 MJ/m^2^ to 67 MJ/m^2^. In addition, the mass loss was significantly delayed after 150 s, indicating enhanced thermal–oxidative stability during combustion. Further analysis suggests that an aromatic-rich char network is formed above 550 °C. The introduction of phenylboronic acid improves char quality through the formation of B–O–C, B–O–P, and B–C crosslinked structures, resulting in a more compact and thermally stable carbonaceous residue. It is therefore inferred that B-APP particles play a positive role in the char-forming process, particularly during the later stage of combustion.

The advantages and unavoidable limitations associated with different boron–APP synergistic strategies are summarized in Table 4. Although these modification strategies enhance flame retardancy by promoting protective char formation, they inevitably involve trade-offs among processing feasibility, interfacial compatibility and scalability.

#### 3.1.3. Metallic-Containing Compounds

Transition metals such as Fe, Al, Ni, Cu, and Mo, in the form of salts, oxides, or complexes, are commonly used as synergistic flame retardants with APP. These metal compounds catalyze crosslinking reactions in IFR systems via dehydration and oxidation. For instance, metal salts such as aluminum hypophosphite [109], zinc sulfate, and nickel sulfate have been shown to enhance flame retardancy in both gas and condensed phases when combined with APP [110]. Regarding metal oxides, Gao et al. [111] and Wang et al. [112] observed that low concentrations of Fe_2_O_3_ or Al_2_O_3_ reduce heat release from the polymer substrate during the initial combustion stage. The catalytic effect of these metal oxides promotes deamination and dehydration crosslinking reactions with APP, forming bridging bonds. Although limited in number, these bridging bonds enhance the stability and graphitization of the char structure while reducing the amount of pyrolysis products. In contrast, Sb_2_O_3_ has a lower melting point (≈656 °C) than Fe_2_O_3_ and Al_2_O_3_ and melts upon heating, coating the substrate surface and creating a physical barrier that blocks oxygen access. Sb_2_O_3_ achieves synergistic flame retardancy with APP through endothermic reactions, complementing the catalytic effect of transition metal oxides.

Numerous recent studies have explored the use of diverse MOFs as synergistic flame retardants and have shown that they can improve flame retardancy and substrate compatibility. This has become one of the most prominent research fields in recent years. Table 5 summarizes the effects of various MOFs and APP on the relevant properties of organic substrates, demonstrating a positive role in improving both mechanical and combustion performance. Specifically, application studies involving MOFs such as ZIF-8 [113,114], ZIF-67 [115], CoZn-ZIF [116], and Ni-MOF [117] have demonstrated that the presence of organic ligands within MOFs, coupled with their excellent compatibility with organic substrates, mitigates the deterioration of the original mechanical properties of substrate caused by the addition of APP-based flame retardants [117]. Even at low loadings (1 wt% to 10 wt%), MOFs exhibit significant anti-dripping effects. Their metal ions catalyze the decomposition of both the substrate and APP, promoting early carbonization and enhancing crosslinking and char formation [118,119]. The dense char layer inhibits heat release, protects the polymer from melt droplets, and suppresses smoke and toxic gas emission. Furthermore, the active metal oxide species generated during MOF thermal decomposition can adsorb toxic CO and catalyze its conversion into less harmful products such as CO_2_, thereby reducing the release of toxic gases.

Despite their effectiveness, most of these systems rely on heavy metals (e.g., Co, Ni), which raise concerns regarding environmental pollution and long-term ecological risks. Consequently, there is an increasing shift toward green and bio-based alternatives. For instance, Song et al. [126] developed a bio-based flame retardant, calcium gluconate (CG)-modified APP(CG@APP6), via coordination between Ca^2+^ and APP. With only 6 wt% loading in EP (EP/CG@APP6), the composite achieved a UL-94 V-0 rating. Moreover, compared with EP/APP6, EP/CG@APP6 exhibited a 70.5% reduction in pHRR and a 50.0% decrease in pSPR. This improvement is attributed to a synergistic gas-phase and condensed-phase mechanism, which facilitates the formation of a more continuous and compact char layer, thereby effectively inhibiting heat transfer and combustible gas release. Additionally, the hydroxyl-rich and Ca^2+^ functionalized surface of CG@APP enhances interfacial compatibility through hydrogen bonding and coordination interactions, leading to increases of 18.2%, 4.5%, and 9.1% in tensile, flexural, and impact strengths, respectively, compared to neat EP. This trend toward sustainable design is further exemplified by Wang [77], who constructed a bio-inspired polydopamine coating on APP via metal ion coordination.

The advantages and challenges of different metal-containing APP-based IFRs are summarized in Table 6. Traditional metal salts and metal oxides mainly enhance flame retardancy through the catalytic promotion of char formation. In addition, metal-coordination-based modification provides a versatile platform for tailoring the structure and properties of APP through the rational selection of metal ions and organic ligands, enabling diverse synergistic flame-retardant effects. However, previous studies have primarily focused on flame retardancy and mechanical properties, while the influence of metal components on hygroscopicity, corrosion behavior, and long-term reliability remains insufficiently understood. Therefore, future development should prioritize environmentally benign metal-coordination systems with improved stability, scalability, and practical applicability.

### 3.2. Structure Synergistic Systems

#### 3.2.1. Nanostructures

Nanostructured materials, such as POSS and carbon nanotubes, have been incorporated into APP to fabricate micro–nano composite flame retardants. These nanoscale additives enhance the thermal stability and flame retardancy of polymers through four primary mechanisms: (1) acting as heat barriers and redistributing heat, thereby delaying thermal degradation; (2) forming protective char layers on polymer surfaces during combustion; (3) catalyzing and promoting char formation; and (4) increasing polymer melt viscosity [127]. The nanoscale effect and exceptionally large specific surface areas of these additives also promote volume expansion, further contributing to flame retardancy.

POSSs are organic–inorganic hybrid materials that can be readily functionalized with diverse chemical groups. With the development of POSS-based nanocomposites, researchers have combined functionalized POSS with APP-based IFR systems to construct Si/P/N synergistic flame-retardant systems. For instance, Tang et al. [128] and Gizem Turgut et al. [129] incorporated different functionalized POSS derivatives into APP/PP systems. Their results demonstrated enhanced char-forming ability and improved flame-retardant performance, together with improved mechanical properties. In particular, the barrier effect was significantly strengthened, as the thermal insulation performance increased with increasing octaphenyl POSS (OPS) loading. POSS derivatives bearing reactive functional groups, such as amine-propyl isobutyl POSS (A-POSS), trisulfonic acid propyl POSS (TS-POSS), and octamine-propyl isobutyl POSS (OA-POSS), exhibited the most pronounced synergistic effects with APP-based intumescent systems. Notably, significant synergistic effects were still observed even at low POSS loadings (0.5 wt%). Among them, the highest char yield was achieved in the presence of TS-POSS, which can be attributed to its acidic functionality that promotes char formation. It has been suggested that the incorporation of POSS nanoparticles enhances char yield and improves char stability through the in situ formation of a ceramic-like protective layer on the char surface, thereby delaying the degradation and collapse of the protective barrier. Specifically, OA-POSS and TS-POSS improved the fracture elongation and impact strength of the composites.

Federico Carosio [130] employed layer-by-layer (LBL) self-assembly to alternately deposit APP and octylammonium POSS onto acrylic fiber fabric. The resulting APP/POSS coatings enhanced flame retardancy: APP promoted the formation of acrylic carbonates, while POSS acted as a thermal insulator to inhibit heat transfer. Yang et al. [131] proposed using octahedral octavinyl POSS (OV-POSS) nanoparticles as flame-retardant synergists and compatibilizers in PP. Owing to electrostatic adsorption driven by the nanoscale effect and the exceptionally large specific surface area, OV-POSS nanoparticles firmly adhered to the flame-retardant powder particles, exerting a compatibilizing effect. Collectively, these studies demonstrate that POSS can significantly enhance flame-retardant and mechanical properties when combined with APP through multiple mechanisms, including char promotion, thermal insulation, and compatibilization.

Jia et al. [132] used 3-aminopropyltriethoxysilane to modify the surface of APP and blended it with carboxylated multi-walled carbon nanotubes for application in high-impact PS. The resulting network-like char layer enveloped polymer melt droplets, inhibiting dripping while providing heat insulation and a physical barrier against volatile species and smoke. In another study [127], halloysite nanotubes were incorporated with APP into EP. The resulting char layer after combustion exhibited a dense structure, significantly reduced pHRR, and had excellent tensile properties. Halloysite nanotube particles physically reinforced the foamed carbon by providing an aluminosilicate framework, while the nanotubes with high aspect ratios bridged and formed networks to enhance the stability of the foamed char at elevated temperatures [128].

In summary, POSS, as an organic–inorganic hybrid building block with a well-defined cage structure, facile functionalization, and excellent thermal stability, and carbon nanotubes with a unique one-dimensional nanostructure and high aspect ratio, have both demonstrated effective synergistic flame-retardant performance in APP-based systems at low loading levels, showing broad application prospects. However, their practical applications are still limited by certain drawbacks, such as relatively high cost, limited large-scale availability, and potential aggregation-induced deterioration of dispersion at higher loadings.

#### 3.2.2. Layered Structures

Layered materials have attracted extensive attention as synergistic components in APP-based flame-retardant systems due to their two-dimensional morphology, high aspect ratio, and excellent barrier properties. These structural features enable the construction of compact and thermally stable char layers, thereby effectively suppressing heat transfer, oxygen diffusion, and the release of flammable volatiles during combustion. Representative layered systems include layered silicates (e.g., montmorillonite, MMT), layered double hydroxides (LDHs), mica, and emerging two-dimensional nanomaterials such as MXenes and graphene derivatives.

Among layered silicates, montmorillonite (MMT) has been widely investigated as a synergistic additive for APP. Various strategies, including physical blending and interfacial engineering, have been developed to improve its dispersion and compatibility within polymer matrices. For instance, Jiang et al. [133] designed PU microcapsules (PU@A-M) via ion-exchange interactions between APP and MMT followed by interfacial polymerization, achieving co-encapsulation of both components. In contrast, physical blending systems have also been widely reported [134], although the dispersion state of MMT remains a key factor governing flame-retardant efficiency. In PP-based IFR systems, the incorporation of a small amount of organo-modified MMT significantly enhances flame retardancy, whereas excessive loading leads to particle aggregation and deteriorated mechanical performance due to poor dispersion.

The synergistic mechanism of MMT with APP is primarily attributed to its physical barrier effect and interfacial interactions. The high-aspect-ratio layered structure generates a tortuous pathway that retards the transport of heat, oxygen, and degradation products. Interactions such as hydrogen bonding and physical entanglement increase the melt viscosity, limiting polymer chain mobility and stabilizing the condensed phase. During combustion, APP decomposition generates polyphosphoric acid, which promotes dehydration and carbonization of the polymer matrix. Simultaneously, interfacial interactions between phosphate species and silicate layers facilitate the formation of a compact and crosslinked carbonaceous network. In addition, the relatively low surface energy of MMT promotes its migration toward the char surface, further contributing to char densification and structural integrity [133,134,135].

LDHs exhibit a distinct flame-retardant behavior compared to layered silicates. Upon thermal decomposition, LDHs release nonflammable gases such as H_2_O, NH_3_, and CO_2_, which dilute combustible volatiles and reduce oxygen concentration near the polymer surface. Concurrently, LDHs are transformed into mixed-metal oxides that serve as inorganic scaffolds, reinforcing the char layer and effectively suppressing crack propagation and structural collapse [127,136].

Mica is a naturally occurring layered aluminosilicate that has also been employed as a synergist in APP-based systems. Li et al. [137] demonstrated that incorporating mica into recycled PE systems containing APP and guanidine cyanurate significantly reduced char porosity and crack formation. Its rigid layered architecture enhances the mechanical strength and sealing capability of the residual char, thereby improving the integrity of the protective barrier at elevated temperatures. Furthermore, mica reacts with phosphates and phosphorus oxides generated during APP decomposition at high temperatures, producing a coherent and dense ceramic-like microstructure. The resulting ceramified residue exhibits enhanced mechanical strength and structural integrity, which improves the stability of the protective barrier during combustion [138,139].

In recent years, emerging two-dimensional nanomaterials have further expanded the design space of APP-based flame-retardant systems owing to their ultrathin layered structures, high specific surface areas, and excellent barrier properties. Among them, MXenes and graphene have attracted considerable attention as efficient synergistic additives.

Wang et al. [140] developed a multifunctional flame-retardant phase-change composite incorporating APP and MXene. During combustion, MXene nanosheets act as an effective physical barrier to retard heat and mass transfer, while the partial oxidation of MXene generates TiO_2_, which further promotes char formation and enhances the thermal stability of the protective layer. Consequently, the synergistic interaction between MXene and APP results in the formation of a dense inorganic–organic hybrid char enriched with Ti-, P-, and N-containing species, thereby significantly improving smoke suppression and thermal shielding performance [140,141].

Compared with MXenes, graphene possesses excellent mechanical strength and barrier capability but suffers from severe restacking and aggregation in polymer matrices because of the strong π–π stacking interactions and van der Waals forces between adjacent nanosheets [142]. Such aggregation substantially deteriorates its dispersion and limits its synergistic flame-retardant efficiency. Therefore, surface modification is commonly employed to improve the interfacial compatibility of graphene-based materials with polymer matrices. For example, modified graphene oxide (MGO) exhibits excellent synergistic effects with APP, where a low loading (0.1 wt%) effectively reduces polymer flammability and toxic gas evolution while promoting the formation of a denser and more compact residual char through the synergistic action of APP and MGO [143].

Similarly, Shi et al. [50] modified APP with vinyltrimethoxysilane (Si-171) to prepare APP@Si-171, which was subsequently combined with graphene in high-impact polystyrene (HIPS). The silane modification improved the interfacial compatibility between APP and graphene, leading to more homogeneous filler dispersion, enhanced thermal stability, and reduced heat release. However, excessive graphene loading causes nanosheet aggregation, which weakens the barrier effect and consequently reduces the overall flame-retardant performance. The superior flame-retardant performance of graphene-based APP systems relies on a well-dispersed two-dimensional network through appropriate surface modification and controlled filler loading, thereby maximizing the barrier effect and condensed-phase reinforcement.

Table 7 summarizes the representative loading ranges of typical layered materials in APP-based flame-retardant systems. The appropriate loading varies with the polymer matrix, formulation, and processing conditions. Generally, MMT exhibits effective barrier effects at low loading levels but is prone to aggregation at higher levels. LDHs provide additional flame-retardant effects through nonflammable (H_2_O, CO_2_) gas dilution and metal oxide formation; however, the collapse of their layered structure during combustion weakens their barrier effect after thermal decomposition. Mica enhances char integrity through its rigid structure and ceramic-forming ability, although relatively high loading is usually required [144]. In contrast, MXenes and graphene derivatives achieve efficient barrier reinforcement at low loadings, but their practical application is restricted by high cost and nanosheet aggregation. Overall, layered materials synergize with APP mainly through barrier effects, condensed-phase reinforcement, and the formation of inorganic or ceramic-like protective structures. Future research should focus on scalable surface modification and interface engineering to optimize the balance among flame retardancy, dispersion, and processing performance.

#### 3.2.3. Cyclic Structures

The first-generation IFR system, consisting of APP as the acid source, PER as the carbon source, and MEL as the blowing agent, suffers from several limitations, including high additive loading, poor water resistance, limited thermal stability, and migration of low-molecular-weight PER and MEL during processing and service. To overcome these drawbacks, cyclic nitrogen- and phosphorus-containing compounds have attracted considerable attention as synergistic charring agents for APP due to their rigid molecular structures, enhanced thermal stability, and abundant reactive sites.

**Figure 8:**
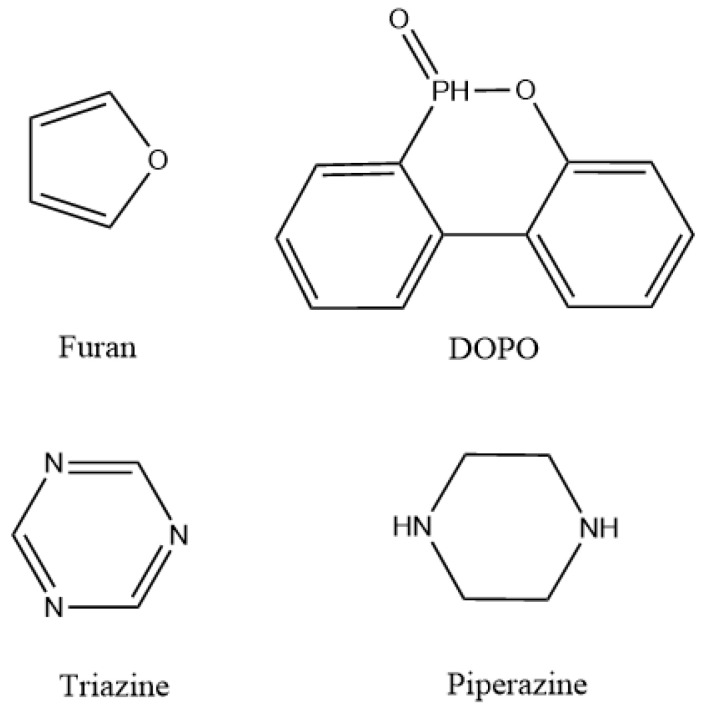
Representative cyclic structures used as synergistic charring agents in APP-based flame-retardant systems.

Representative cyclic structures investigated as APP synergists include triazine-, piperazine-, furan-, and DOPO-based compounds (Figure 8). Compared with conventional PER/MEL systems, these cyclic structures readily undergo cyclization or self-crosslinking during combustion. In combination with phosphoric and polyphosphoric acids generated from APP decomposition, they promote the formation of compact and highly crosslinked char layers, thereby enhancing condensed-phase flame-retardant performance.

Liu et al. [153] described piperazine-based charring agents, including a triazine–piperazine compound containing Schiff base functionalities. During combustion, these compounds undergo in situ cyclization and self-crosslinking, forming elastic and thermally stable crosslinked char layers with a wrinkled and compact morphology, thereby improving the integrity of the protective barrier.

In addition to forming condensed-phase char, phosphorus-containing cyclic structures can also contribute to gas-phase flame inhibition. Lim et al. [154] synthesized a phenyl-DOPO derivative as a synergistic additive for APP, which effectively scavenges H• and OH• radicals in the gas phase, while APP- and DOPO-derived acids catalyze dehydration and carbonization in the condensed phase, leading to enhanced flame-retardant performance.

Furan-containing compounds have also been explored as APP synergists; the furan ring undergoes thermal polymerization to form stable carbonaceous networks. In the presence of APP-derived phosphoric acids, dehydration and crosslinking reactions are further promoted, facilitating the formation of compact char structures with improved thermal stability [155]. Similarly, Liu et al. [156] reported APP/melamine polyphosphate(MPP)/tris(2-hydroxyethyl)isocvanurate(THEIC) systems, where phosphoric acids catalyze the esterification and carbonization of triazine-containing structures, resulting in thermally stable char formation and improved mechanical properties.

Chen et al. [157] synthesized a multifunctional charring agent (PDTBP) that decomposes to generate phosphorus-containing species and nonflammable gases (NH_3_, H_2_O, CO_2_), promoting crosslinking, intumescence, and the formation of a compact expanded char layer. Wang et al. [158] designed a triazine–pentose alcohol-based system that forms dense and mechanically robust char. At elevated temperatures, triazine units can transform into graphitic carbon nitride, while APP promotes carbonization, jointly reinforcing the condensed-phase protective layer [159].

Despite these advances, most cyclic charring agents still require multistep synthesis and organic solvents and have relatively high production costs, which limits their large-scale application. Therefore, developing simple, sustainable, and cost-effective cyclic carbonizing agents remains a key challenge.

## 4. Conclusions and Future Outlook

APP is a halogen-free flame retardant that is widely used due to its high flame-retardant efficiency, low toxicity, low smoke emission, and cost-effectiveness. Its phosphorus-rich structure promotes the formation of stable and compact char layers during combustion, which effectively enhances the fire resistance of a wide range of polymer systems, supporting its extensive industrial applications.

Despite these advantages, several intrinsic limitations remain, particularly related to its hygroscopic nature, limited interfacial compatibility with hydrophobic polymers, and aggregation tendency, which can adversely affect the long-term performance of flame-retardant composites. To address these issues, surface modification strategies have been widely developed to improve hydrophobicity, dispersion and interfacial adhesion. However, most current approaches rely on multi-step processes or complex reaction systems, which hinder large-scale industrial application. Therefore, future research should focus on designing one-step, solvent-free, or in situ modification strategies that are both scalable and economically viable.

In addition, synergistic systems incorporating APP with nanostructured fillers (e.g., LDH, clay, and silica), metal–organic frameworks (MOFs), and bio-based carbon sources have shown promising improvements in char formation and thermal stability. Nevertheless, the interfacial compatibility, long-term dispersion stability, and long-term reliability of these hybrid systems remain insufficiently understood. Future efforts should therefore emphasize interface engineering and dispersion control at the nanoscale, enabling more stable and efficient synergistic flame-retardant architectures.

From a mechanistic perspective, although significant progress has been made in understanding the condensed-phase and gas-phase flame-retardant actions of APP-based systems, a unified structure–property–performance relationship is still lacking. Advanced characterization techniques combined with simulation methods (e.g., molecular dynamics and reactive modeling) are expected to provide deeper insights into degradation pathways and synergistic mechanisms.

Furthermore, future research on APP-based flame-retardant systems should adopt more application-oriented fire safety evaluation strategies rather than relying solely on conventional laboratory-scale tests. Currently, LOI, UL-94, and cone calorimetry are the most commonly used methods for evaluating flame retardancy and fire behavior. However, the critical fire safety evaluation parameters vary significantly among different application fields. For example, aerospace materials require evaluation of heat release, smoke density, toxic gas emission (e.g., HF, HCl, HCN), and flammability (e.g., 12 s/60 s vertical burning test) according to FAR 25.853 [160], while rail transportation materials are assessed based on heat release, smoke density, glow-wire test parameters, and toxic gas emissions specified by EN 45545-2 [161]. Construction materials mainly focus on reaction-to-fire classification according to EN 13501-1 [162], whereas automotive and electrical/electronic applications emphasize horizontal burning rate and glow-wire test parameters according to FMVSS 302 [163] and IEC 60695 series [164,165], respectively. Nevertheless, systematic investigations of smoke density, smoke toxicity, and toxic combustion products remain relatively limited in APP-based flame-retardant studies. Therefore, future studies should focus on improving the consistency between fire safety evaluations and practical application requirements, providing guidance for the rational selection and application of different APP-based intumescent flame-retardant systems.

Sustainability represents another important challenge for the future development of APP-based flame-retardant systems. Although APP is considered a relatively environmentally friendly halogen-free flame retardant, the recyclability of APP-containing polymer composites and the influence of APP-based additives on polymer degradation and recycling behavior remain largely unexplored. For biodegradable and bio-based polymers, the effects of APP on hydrolysis behavior and microbial degradation require further systematic investigation. Therefore, future efforts should focus on developing recyclable, biodegradable, and environmentally compatible APP-based flame-retardant systems while considering their impacts on polymer circularity and life-cycle sustainability.

Finally, in terms of industrial translation, future development should prioritize processability, recyclability, and environmental sustainability, particularly in large-scale polymer processing systems. The design of multifunctional flame-retardant systems that simultaneously provide mechanical reinforcement, smoke suppression, and environmental friendliness represents a key direction for next-generation APP-based materials.

## Figures and Tables

**Table 1:** Representative modification strategies of APP and their corresponding mechanisms, advantages, and limitations.

Interaction Mechanism		Modifier Type	Advantages	Limitations	References
Covalent coupling modification		Silane coupling agents	Provides enhanced interfacial stability; enables effective organic–inorganic surface functionalization of APP; improves dispersion within polymer matrices; reduces hydrophilicity	Potential partial degradation or alteration of APP structure under harsh reaction conditions	[50,51,52,53,54,55]
Non-covalent interactions	Amino-functional and electrostatic interactions	Organic compounds with amino groups	Mild and facile processing conditions (typically room temperature); preserves intrinsic structure of APP; effectively reduces water solubility	Limited structural stability of the modified layer; potential reduction in thermal stability under prolonged heating	[56,57,58,59]
	Hydrogen-bonding-dominated interactions	Hydrogen-bonding-rich organic molecules	Simple preparation process under mild conditions; avoids destruction of APP crystal structure; improves surface compatibility to a certain extent	Poor long-term water resistance; limited enhancement in thermal stability	[60,61,62,63,64,65,66,67,68,69]
	Multiple interactions (shell-forming reaction system)	In situ shell-forming systems	Significantly improves dispersion stability and reduces water uptake of APP; enables surface protection through continuous coating layers	Complex preparation procedures; limited reproducibility and process controllability	[70,71,72,73]
Metal coordination		Metal ions and coordination ligands	Highly tunable structure with strong design flexibility; enables synergistic catalytic char formation and structural reinforcement	Relatively high cost; complex coordination systems and synthesis routes	[74,75,76,77,78,79]

**Table 2:** Performance of APP-based flame-retardant systems with kaolinite [88,89,90,91,92].

Additive Content	Total APP-Based Flame Retardant Content	Matrix	Performance Compared with Single APP-Based IFR System					
			LOI (%) and Relative Increase	UL-94 Rating	pHRR (kW/m^2^)	THR (MJ/m^2^)	TSP * (m^2^)	Tensile Strength
1%	25%	PP	39.5% (+16.0%)	V-0, no change	—	—	—	−33%
2%	28%	EP	30.3% (+6.6%)	V-0, no change	−12%	−3%	−45%	+2%
3%	15%	Unsaturated polyester resins	27.3% (+4.2%)	—	−6%	−3%	−15%	+5.7%
2%	20%	EVA	28.6% (+14.9%)	V-2, no change	−22.9%	−3.8%	−18.6%	+3.9%

* Total smoke production.

**Table 3:** Summary of silicon-based systems and their synergistic flame-retardant mechanisms in APP-based composites.

Type	Representative Examples	Advantages	Limitations	Dominant Mechanism
Silicate minerals	Kaolinite, vermiculite	Low loading; strong condensed-phase reinforcement; effective ceramic-like char formation; good smoke suppression	Poor interfacial compatibility; mechanical property deterioration	Physical barrier and ceramic char reinforcement
	Wollastonite, basalt powder	Low cost	High loading; poor interfacial compatibility	
SiO_2_		Low loading; thermal insulation; low loading efficiency improved thermal stability	Weak char-forming ability; morphology-dependent efficiency; limited flame-retardant effectiveness	Thermal shielding and physical barrier effect
Organosiloxane modifiers	Silane coupling agents, sol–gel coated APP, polysiloxane shells	Improved interfacial compatibility; enhanced dispersion; improved water resistance and mechanical properties; better char integrity	Complex synthesis; cost and scalability issues; performance sensitive to coating quality	Interfacial engineering and Si–P synergistic char formation

**Table 4:** Comparison of boron–APP synergistic strategies: advantages, limitations.

Boron–APP Synergistic Strategy	Advantages	Limitations
Physical blending of boron compounds with APP	Simple processing procedure; low cost; easy industrial implementation	Weak interfacial interactions between additives and polymer matrices; poor compatibility and dispersion stability; possible migration of additives during long-term use
Incorporation of boron into charring agents to enhance synergistic interactions with APP	Enhances char formation and barrier performance by generating B–O–P structures	Increases formulation complexity; excessive addition may negatively affect melt processability and mechanical properties
Surface modification of APP using boron- and amino-functionalized compounds	Improves interfacial adhesion, dispersion stability, and moisture resistance of APP; reduces compatibility issues with polymer matrices	Requires additional modification processes; increased preparation cost and complexity; optimization of surface modification degree is necessary
Synthesis of boron-containing APP derivatives.	Provides stronger chemical coupling between boron species and phosphate groups; facilitates formation of stable B–O–P/B–O–C structures during combustion; improves synergistic efficiency	Complex synthesis routes; limited scalability; high production cost may restrict practical applications

**Table 5:** Synergistic flame-retardant performance of MOFs/APP systems: metal centers, ligands, preparation methods, and property improvements.

Metal Types		Ni [117,120]		Cu [121,122]		Co [123,124]		Fe [125]
Organic ligand		DHTAa	PPHDIb	H2BDC- NH2c	BTAd	2MIe	MDAf	Tris-PDAg
Methods of combining APP with MOFs		MCh	PDi, 66% APP	Silane coupling agent	PD, 98%APP	MCh	PDi, 75%APP	PDi, 68%APP, 23%PER
Flame-retardant dosage		5%	5%	9%	8%	30%	6%	22%
Application substrate		EP	PLA	EP	TPU	Corn Stalk/PP	TPU	PS
Improvement vs. APP alone	LOI relative increase	+6.9%	+27.6%	+2.4%	+3.8%	+11.8%	+10.2%	+3.3%
	UL-94 vertical burning classification	NR to V-1	V-2 to V-0	NR to V-0	V-1 to V-0	V-0, no change	V-2 to V-0	NR to V-0
	Tensile strength	+45.8%	+8.1%	+10.9%	—	+36.8%	+18.5%	+5.2%
	Bending strength	+6.87%	—	—	—	+11.1%	—	+5.6%
	Elongation at break	+22.68%	+15.0%	+11.8%	—	—	+30.3%	—
	Impact strength	+11.6%	+14.2%	—	—	—	—	+29.4%
	pHRR (kW/m^2^)	—	—	—	−14.1%	−38.8%	−4.1%	−49.3%
	THR (MJ/m^2^)	—	—	—	−64.2%	−1.5%	−3.1%	−21.0%

a. 2,5-Dihydroxyterephthalic acid; b. α-Phenyl-N-(2-propyl-2-hydroxymethyl-1, 3-dihydroxy)-Imine; c. 1,3,5-benzene tricarboxylic acid; d. 1,3,5-benzene tricarboxylic acid; e. 2-methylimidazole; f. 4,4-Diaminodiphenyl methane; g. carbonization groups (reacted by tris(hydroxymethyl) aminomethane and 1,4-piperazine dialdehyde); h. metal coordination; i. physical blending.

**Table 6:** Comparison of metal-containing APP-based flame-retardant systems: advantages, limitations and challenges.

Metal-Containing APP-Based IFRs	Advantages	Limitations and Challenges
Metal-containing inorganic compounds (e.g., metal salts and metal oxides)	Low cost and wide availability; promote catalytic dehydration reactions and char formation, facilitate graphitization of carbonaceous residues; improve thermal stability and protective char layer integrity.	Performance strongly depends on metal species and loading levels; excessive inorganic components may cause poor dispersion, reduced mechanical properties, and processing challenges.
MOFs	Tunable metal centers, organic ligands, and diverse coordination environments; efficient catalytic promotion of char formation, melt-dripping suppression, and toxic gas emission reduction at low loading levels; improved interfacial compatibility with organic matrices.	Relatively high cost, complex synthesis procedures, limited scalability, potential environmental concerns associated with Co/Ni-based systems, and limited long-term stability.
Bio-derived metal-containing compounds (e.g., CG)	Renewable bio-derived resources; abundant oxygen-containing functional groups and metal ions; promote catalytic char formation and improve char layer stability; enhance interfacial compatibility with polymer matrices.	Limited studies have been reported on bio-derived metal-containing compounds; further investigation is required on their synergistic mechanisms, hygroscopicity, and long-term reliability.

**Table 7:** Representative loading ranges of typical layered synergists in APP-based flame-retardant systems.

Layered Material	Typical Loading (wt%)	Reference
MMT	0.5–3%	[131,132,133,140,141]
LDHs	1–50%	[127,136,145,146,147,148]
Mica	2–33%	[136,137,149]
MXene	1–10%	[138,139,144,150]
Graphene	0.1–12%	[48,151,152]

## Data Availability

No new data were created or analyzed in this study. Data sharing is not applicable to this article.
